# Effect of the Addition of Biobased Polyols on the Thermal Stability and Flame Retardancy of Polyurethane and Poly(urea)urethane Elastomers

**DOI:** 10.3390/ma14071805

**Published:** 2021-04-06

**Authors:** Kamila Mizera, Kamila Sałasińska, Joanna Ryszkowska, Maria Kurańska, Rafał Kozera

**Affiliations:** 1 Faculty of Materials Science and Engineering, Warsaw University of Technology, Woloska 141, 02-507 Warsaw, Poland; joanna.ryszkowska@pw.edu.pl (J.R.); rafal.kozera@pw.edu.pl (R.K.); 2 Department of Chemical, Biological and Aerosol Hazards, Central Institute for Labour Protection—National Research Institute, Czerniakowska 16, 00-701 Warsaw, Poland; kamila.salasinska@ciop.pl; 3 Faculty of Chemical Engineering and Technology, Cracow University of Technology, Warszawska 24, 31-155 Cracow, Poland; mkuranska@indy.chemia.pk.edu.pl

**Keywords:** biobased polyols, polyurethane elastomers, thermal stability, fire behavior, char residues

## Abstract

Due to the current trends in sustainable development and the reduction in the use of fossil fuels (Green Deal strategy and the circular economy), and thus, the increased interest of the polyurethane industry in polyols derived from renewable sources, it is important to study the impact of these polyols on the flammability of new bioelastomers. The goal of this study was to check the influence of biobased polyols, such as tall oil (TO)-based polyols, soybean oil (SO)-based polyol, and rapeseed oil (RO)-based polyol, on the reduction in the burning and fume emissions of polyurethane and poly(urea)urethane elastomers (EPURs and EPUURs). The thermal stability of these materials was tested using thermogravimetric analysis (TGA). In turn, the flame retardancy and smoke emissions were checked using a cone calorimetry test. The released gases were identified using TGA coupled with Fourier transform infrared (FT-IR) spectroscopy (TGA/FT-IR). Moreover, the morphological and structural characteristics of the char residues were characterized using FT-IR and scanning electron microscopy (SEM) with energy-dispersive spectroscopy (EDS). The obtained data were compared to the results received for elastomers produced with petroleum substrates. The addition of biobased polyols led to a reduction in the burning as a result of the formation of char, especially RO polyol. Moreover, the TO and RO polyols increased the thermal stability of the elastomers.

## 1. Introduction

Elastomers, including polyurethane elastomers (EPURs), are polymeric materials that are popular for their excellent combination of chemical and physical properties, coupled with their low cost and excellent processability [1]. EPURs find numerous commercial applications in many industries in the form of adhesives, coatings, textiles, or elements in medical devices, cars, electronics, etc. [2,3,4]. EPURs are an important class of functional polymers whose properties can be designed through the relative composition of the soft segment (SS) and hard segment (HS), which are determined by the molecular chain structures of the constituents [5,6,7]. The SS, which is obtained from the reaction of isocyanate and polyol, contributes to the flexibility and damping properties of an EPUR. In contrast, the HS, which is obtained in the formation of urethane or urea groups from the reaction of isocyanate with a chain extender, contributes stiffness and strength to an EPUR. The segmented structure is the main reason for their excellent properties, which consequently means that EPURs meet the diverse demands of modern technologies [8].

In recent years, plant components have been introduced to polyurethane systems to meet the ideals of sustainable development but this is a significant challenge for chemical companies [9]. The search for sustainable solutions has promoted research into substituting petrochemical materials with renewable feedstocks [10]. There is a hope that renewable materials will allow for the rational use of resources and reduce the negative environmental impact of produced polymers, creating a more sustainable solution for common materials [11]. Renewable resources are of particular interest as an alternative to fossil resources for energy production and as starting materials for industrial chemistry due to the lower cost spectrum. For this purpose, petrochemical polyols, one of the main components of EPUR synthesis, have been replaced by polyols from renewable resources [12]. In EPUR production, the alternative polyol components that are used most often are biopolyols derived from vegetable oils because of the greater availability of their resources. They are most often used for the production of polyurethane foams, although over the past few years, there have been several publications regarding the use of plant polyols for the production of EPURs, including our publications [13,14,15,16,17,18,19]. However, before vegetable oils can be used for the production of polyurethanes, they have to be chemically processed to contain hydroxyl groups. It is possible to convert the unsaturated parts of vegetable oils to hydroxyl groups with chemical treatment [17,18].

It is well known that EPURs display supreme resistance to abrasion, solvent and fatty environments, and weather conditions, such as oxygen, ozone, and sunlight. However, their combustibility and lack of thermal stability increase their overall fire risks [20]. Currently, the flame retardancy of polyurethanes is improved by the addition of flame retardants, mainly phosphor [1,21,22]. Vegetable oils are composed of triglyceride molecules, which are products obtained from one glycerol molecule and three fatty acid molecules. Fatty acids are categorized into saturated and unsaturated fatty acids depending on the type of vegetable oil. Saturated fatty acids do not have double bonds, while unsaturated fatty acids may have one or more of these bonds [23]. According to the available literature [24], the double bonds are the precursors to char. Therefore, studies of EPURs related to the increase in its high resistance to burning in the presence of environmentally friendly flame retardant additives seem to be important [25,26]. The subjects of this study are EPURs and poly(urea)urethane elastomers (EPUURs) made with tall oil (TO)-based polyol, soybean oil (SO)-based polyol, and rapeseed oil (RO)-based polyol, which replaced different substrates. In our previous works, EPUURs with TO polyols as chain extenders demonstrated a higher thermal resistance, rigidity, and reduction in the permanent deformations than the EPURs manufactured with the use of ethylene glycol (EG) [13,19]. In turn, EPURs with SO polyol substitution of petrochemical polyol at 100 wt% had higher thermal and strength properties [15,16]. Furthermore, we observed that the lower molar mass of SO polyols led to greater hardness, higher abrasion resistance, and a greater ability to dampen vibrations. In other works [17,18,19], we showed that higher annealing temperatures resulted in higher abrasion resistances and did not change the thermal stability relative to a reference EPUUR, and that the difference in the structures of RO polyols had an impact on the thermal and mechanical properties. However, the question of the impact of vegetable oil on the burning of EPURs remains unresolved. The purpose of this work was to check the influence of the mentioned biobased polyols on the burning behavior and fume emission of tested EPURs and EPUURs. Moreover, the thermal stability of the polymers and the identification of the released gases were determined.

## 2. Experimental

### 2.1. Materials

To produce EPURs and EPUURs, the following components were used: poly(ethylene adipate) (PEA) with the trade name Polios 60/20, with molar masses of 1906 g/mol and 2318 g/mol (Purinova, Bydgoszcz, Poland); 4,4′-diphenylmethane diisocyanate (MDI) (Sigma-Aldrich, Poznan, Poland); ethylene glycol (EG), with a molar mass of 62.07 g/mol (Chempur, Piekary Slaskie, Poland); dicyandiamide (DYDI), with a molar mass of 84 g/mol (Emerald Performance, Vancouver, CA, USA).

The TO-based polyol was prepared by the Latvian State Institute of Wood Chemistry. The biopolyol based on TO with an acid value of 190 mgKOH/g and 20% free rosin acids was prepared via amidization with diethanolamine (DEA) [13]. The used SO-based polyol had a molar mass of 1445 g/mol and a functionality of 3.34 (MN, USA) [15]. The RO-based polyol with hydroxyl value 58 mgKOH/g and acid value 1.83 mgKOH/g was prepared by the Faculty of Chemical Engineering and Technology, Cracow University of Technology, Cracow, Poland [19]. The synthesis methods of the used biobased polyols were described in our earlier publications mentioned above.

Elastomers with the tall-oil-based polyol:

The polyurethane and poly(urea)urethane elastomers (EPUR_1,5 and EPUUR_TO) were produced with the use of TO-based polyol as a chain extender.

The melted PEA was degassed for 1 h at 150 ± 5 °C and 6 hPa with intense stirring. Then, after the PEA was cooled to 60 ± 3 °C, the MDI was added and stirred for 5 min. After that, the TO polyol or EG was added and stirred for the next 5 min.

Elastomers with the soybean-oil-based polyol:

The polyurethane elastomers (EPUR_2,5 and EPUR_SO) were produced using a one-step method with the SO-based polyol as the main polyol.

The SO polyol was degassed for 3 h at 100 ± 5 °C and 6 hPa with intense stirring. After that time, the SO polyol was cooled to 60 ± 3 °C, the MDI was added, and the entire mixture was stirred for the next 10 min. Then, the EG was added and stirred for another 10 min.

Elastomers with the rapeseed-oil-based polyol:

The poly(urea)urethane elastomers (EPUUR and EPUUR_RO) were produced using a one-step method with 50 wt% RO-based polyol.

At first, PEA with DYDI was melted and degassed for 1.5 h at 150 ± 5 °C and 6 hPa with intense stirring. Then, the mixture was cooled to 80 ± 3 °C, the RO polyol was added, and the entire mixture was stirred for 30 min. After that, the mixture was cooled to 60 ± 3 °C, MDI was added, and stirred for another 10 min.

All of the mixtures were poured into a duraluminium mold and annealed for 18 h at 110 °C. The samples were seasoned under ambient conditions for 14 days. The characteristics of the materials are summarized in Table 1.

### 2.2. Methods

The thermogravimetric analysis (TGA) was done using a Q500 analyzer (TA Instruments, New Castle, DE, USA). Samples of about 10 mg were heated from room temperature to 800 °C with a heating rate of 10 °C/min. The tests were carried out in a nitrogen and air atmosphere with a flow rate of 90 mL/min.

Cone Calorimeter (CC) (Fire Testing Technologies, East Grinstead, UK) tests were performed to investigate the burning behavior of the EPURs and EPUURs. The specimens (100 mm × 100 mm × 4 mm) were placed in an aluminum tray and irradiated horizontally at a heat flux of 35 kW/m^2^. The procedure of the tests was performed with the norm [27].

The char residues obtained from the cone calorimetry were tested using scanning electron microscopy (SEM)with energy-dispersive spectroscopy (EDS) acquired on a Hitachi SU70 (Hitachi High-Tech Corporation, Chiyoda-ku, Tokyo, Japan) under high-vacuum conditions at an acceleration voltage of 15 kV.

The residual chars were also measured using a Fourier transform infrared spectroscopy (FT-IR) Nicolet 6700 spectrometer (Thermo Fisher Scientific, Waltham, MA, USA). The powdered samples were mixed with KBr and pressed into pellets. The analysis was performed in the spectral range of 400–4000 cm^−1^ and a resolution of 4 cm^−1^.

Gas-phase analyses were carried out in a TGA Q500 (TA Instruments, New Castle, DE, USA) coupled with an FT-IR Nicolet 6700 spectrometer (Thermo Fisher Scientific, Waltham, MA, USA) using 64 counts. The 15 mg samples were heated from room temperature to 860 °C with a heating rate of 20 °C/min in air with a flow rate of 90 mL/min. The FT-IR gas cell was held at 240 °C and the temperature of the transfer line was 250 °C. The spectral range of the analysis was 400–4000 cm^−1^ and resolution of 4 cm^−1^.

## 3. Results and Discussion

### 3.1. Thermal Properties

**Figure 1:**
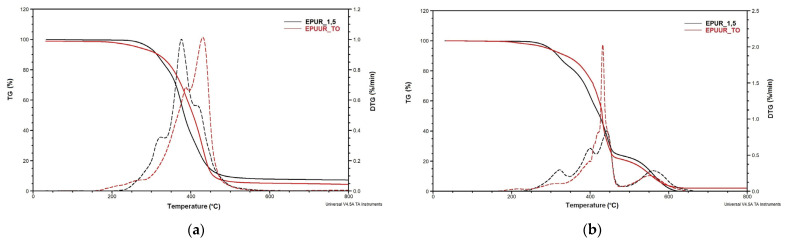

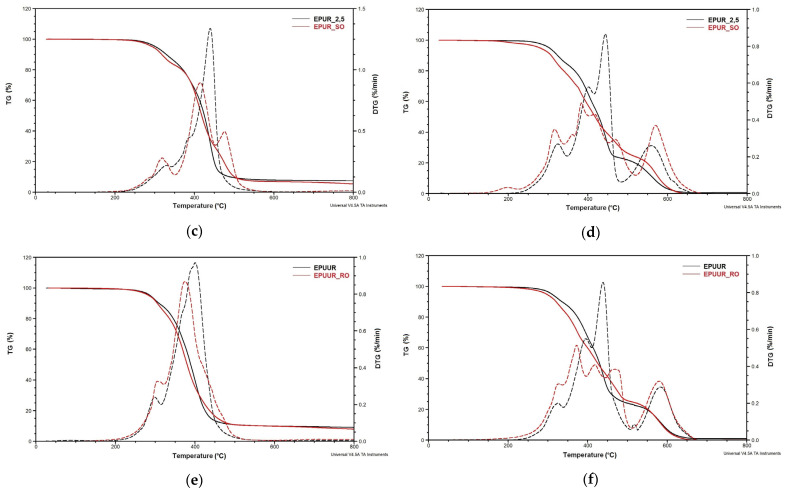
TG (weight) and DTG (derivative weight) curves of EPUR_1,5 (**a**), EPUUR_TO (**b**), EPUR_2,5 (**c**), EPUR_SO (**d**), EPUUR (**e**), and EPUUR_RO (**f**) in a nitrogen atmosphere (left side) and air (right side).

TGA is a commonly used technique to assess the thermal degradation of polymer materials. The curves for the loss of weight (TG) and derivative of the loss of weight (DTG) in a nitrogen atmospheres and air are shown in Figure 1a–f, while the results are summarized in Table 2 and Table 3.

The thermal decomposition of polyurethanes depends on the number of urethane linkages and the content of the aromatic moieties [28]. The DTG curves in nitrogen and air atmospheres showed five and seven stages, respectively, of decomposition corresponding to the maximum rate of degradation of hard segments (T_1_, T_2_, and T_3_ in air) and soft segments (T_4_, T_5_, T_6_, and T_7_ in both atmospheres). The DTG curves also allowed for the determination of the temperature connected with the mass loss at every step of the decomposition (m_1_–m_7_) (Table 2 and Table 3). The first and second maximum peaks are linked to the decomposition of urethane and urea bonds in rigid segments of polyurethane and poly(urea)urethane [29], where urethane bonds decompose at lower temperatures than urea bonds. The third, fourth, and fifth maximum peaks are the results of degradation of the ester bonds in the soft segments that take place from 380 °C, while the decomposition of the aromatic compounds begins at 480 °C [30].

In a nitrogen atmosphere, five stages of degradation were observed (Table 2). The first stage was observed only for EPUUR and EPUUR_RO and occurred at 298 °C and 304 °C (T_1_), with weight losses (m_1_) of 9.5% and 10.2%, respectively. It was observed that the addition of RO polyol increased this temperature. The second stage of degradation occurred at 319–328 °C (T_2_), with a weight loss of 13.0–18.2% (m_2_), and was observed for EPUR_1,5, EPUR_2,5, and EPUR_SO. The peaks mentioned above occurring up to the temperature of 320 °C are related to the distribution of allophanate, biuret, urethane, and urea bonds in the hard segments [31]. The addition of SO polyol resulted in a decrease in this temperature. The third stage of degradation was observed for EPUR_1,5, EPUUR_TO, and EPUUR_RO in the temperature range of 375–387 °C (T_3_), with a weight loss of 36.9–73.3% (m_3_). This temperature range is connected with the decomposition of the ester bond in the materials. For EPUUR_TO, this was the first step of the degradation process. In this material, there was probably no clear phase separation taking place. This is why the first step of decomposition of EPUUR_TO took place at such a high temperature. The temperature T_3_ in this material is the result of the degradation of urethane, ester, and urea linkages presence in this material [32]. The hydrogen bonds between the –NH groups of urethane bonds and oxygen atoms in the flexible segments, i.e., between the –NH and –CO or –O groups, are the first to break down [28]. The –NH groups of the polar hot melt segments in EPUURs strongly interact with each other and are still connected by hydrogen bonds. Urea bonds have more –NH bonds in their groups than the urethane group [33]. The addition of TO polyol to the elastomer resulted in a delay in the thermal degradation of the material and increased its thermal stability. The fourth degradation stage took place in the temperature range of 400–415 °C (T_4_), with a weight loss of 26.6–76.8% (m_4_), and was observed for EPUR_1,5, EPUR_SO, and EPUUR. This temperature is responsible for the breakdown of ester bonds. At this stage, the EPUUR had the greatest weight loss (Table 2). The last, fifth stage of degradation was observed at 430–476 °C (T_5_), with a weight loss of 21.3–77.3% (m_5_). This step of the degradation process was observed for EPUUR_TO, EPUR_2,5, and EPUR_SO, and the greatest weight loss at this stage was observed for EPUR_2,5. In these materials, the degradation of ester bonds in the soft segments and aromatic compounds was probably observed [34].

Different degradation processes were observed for materials in the air atmosphere (Table 3). As shown in Figure 1b,d,f, the end of the degradation in air atmospheres of petrochemical materials and materials with biobased polyols was similar and took place at 650 °C for material with TO and 660 °C for materials with SO and RO polyols, respectively. The first stage of the degradation process occurred at 199 °C and 216 °C (T_1_) with weight losses of 1.7% and 11.0% (m_1_), respectively, and it was observed for EPUUR_TO and EPUR_SO, which could be related to the volatile products in these materials. The second stage of degradation occurred at 316–341 °C (T_2_) and it was similar to the results from the nitrogen atmosphere. The addition of SO polyol resulted in an increase in this temperature. The EPUUR_TO did not display this stage of degradation. The weight loss during this stage was 8.7–17.9% (m_2_) and it was greater after the addition of biobased polyols. The third stage of degradation was observed only for EPUR_SO and EPUUR_RO, and occurred at 361 °C and 372 °C (T_3_), respectively; the weight losses at this stage were 8.2% and 23.7% (m_3_), respectively. At this stage, the degradation of the remaining hard segments in EPUR_SO and EPUUR_RO continued. The fourth degradation stage took place in the temperature range of 384–419 °C (T_4_) with a weight loss of 14.5–28.4% (m_4_). At this stage, the materials with biobased polyols had a lower weight loss than the reference materials (Table 3). The fifth stage of degradation was observed at 416–444 °C (T_5_), with a weight loss of 18.9–51.7% (m_5_). The highest weight loss at this stage was observed for EPUUR_TO. This stage was not observed for EPUUR_RO. The sixth degradation step was observed only for EPUR_SO and EPUUR_RO at 468 °C and 469 °C, with weight losses of 14.4% and 19.2%, respectively. For these materials, the degradation of the soft segments derived from SO and RO polyols probably had a higher thermal resistance and was reduced at higher temperatures [19]. The last step of the degradation occurred at 550–583 °C (T_7_), with a weight loss of 19.1–24.2% (m_7_). The temperature T_7_ decreased after the addition of TO and RO polyols, but increased for EPUR_SO. Such degradation can be explained by the oxidative reaction of the double bonds in the long fatty acid chains of the SO polyol [30].

Based on the TG curves, the temperature at 5% mass loss (T_5%_) and residue at 800 °C (R_800_) were determined. The initial degradation temperature in a nitrogen atmosphere, defined as the 5% weight loss, decreased after the addition of biobased polyols to the elastomers. Oxidative degradation also showed a decrease in this temperature (Table 3); however, the values, excluding materials with SO polyol, were higher compared to the measurements carried out in nitrogen (Table 2). Despite this, the residue percentage for all materials was higher in the nitrogen atmosphere than in air. In air, the residue at 800 °C reached less than 1% of the initial mass. The only exceptions were EPUUR_TO and EPUUR for which the residues at 800 °C were 2.2% and 1.1%, respectively. In the nitrogen atmosphere, a decrease in residue at 800 °C was observed after the addition of biopolyols (Table 2). In turn, after the oxidative degradation at 800 °C, a larger char residue for EPUUR_TO was observed than for EPUR_1,5 (Table 2). The addition of TO polyol to the elastomer matrix can lead to decomposition at higher temperatures and help create char [35].

### 3.2. Burning Behavior

**Figure 2:**
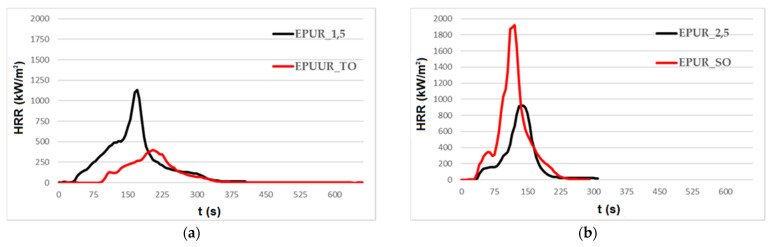

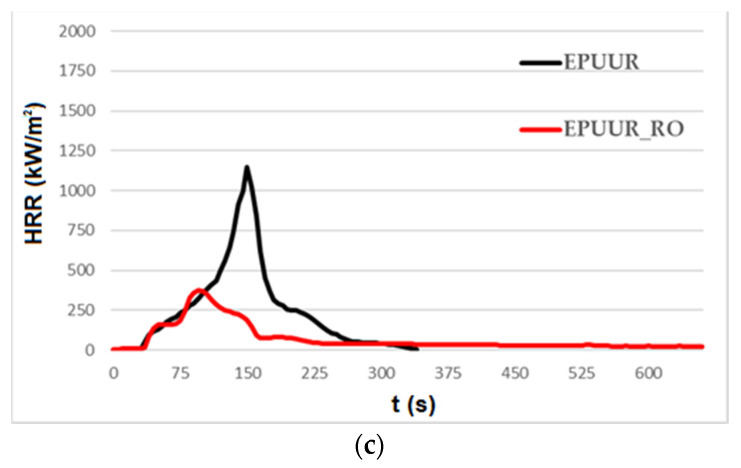
Representative heat release rate (HRR) curves of polyurethane and poly(urea)urethane elastomers with TO polyol (**a**), SO polyol (**b**) and RO polyol (**c**).

Cone calorimetry measurements are commonly applied to investigate polymers’ burning behavior. In Figure 2a–c, representative heat release rate (HRR) curves of EPURs, EPUURs, and their mixes with biobased polyols are juxtaposed and the related data are tabulated in Table 4.

The required time to ignition (TTI) and the flame’s maintenance over the sample surface ranged from 33 to 81 s across the samples; however, no trend was observed. The most important property that determines fire risks is the heat release rate. As can be seen in Figure 2, the HRR curves consisted of more than one stage, where the first was a minor maximum, while the next one demonstrates the peak heat release rate (PHRR). The HRR curve of EPUR_1,5 (Figure 2a) was characterized by a sharp peak with a maximum value that was almost three times higher than that of EPUUR_TO. This characteristic was the result of the wood extractives, such as rosin acid, which influence the burning of materials prepared from them [36]. In turn, in the case of the EPUR_SO, the addition of SO polyol (Figure 2b) led to an increase in the HRR value, and the elastomer had an almost two times higher PHRR than EPUR_2,5. Similar to EPUR_1,5, the maximum peak of HRR for EPUUR_RO was significantly lower than for EPUUR. Consequently, the mean values of the heat release rate (av-HRR), shown in Table 4, were decreased by 64% and 74% in the cases of EPUR_1,5 and EPUUR_RO, respectively, or increased by 180% for EPUR_SO. Similar dependencies were noted for the maximum average rate of heat emission (MARHE) and PHRR/t_PHRR_ which provides information about the possibility for fire growth and spread. The MARHEs of the reference materials were in the range of 354–407 kW/m^2^, while for EPUR_1,5, EPUUR_RO, and EPUR_SO, they were 170, 180, and 613 kW/m^2^, respectively. Even greater discrepancies were noted in the case of PHRR/t_PHRR_, for which the lowest value determined for EPUUR_TO was 2.3 kW/m^2^s, and the highest for EPUR_SO, which was as high as 23.5 kW/m^2^s.

**Figure 3:**
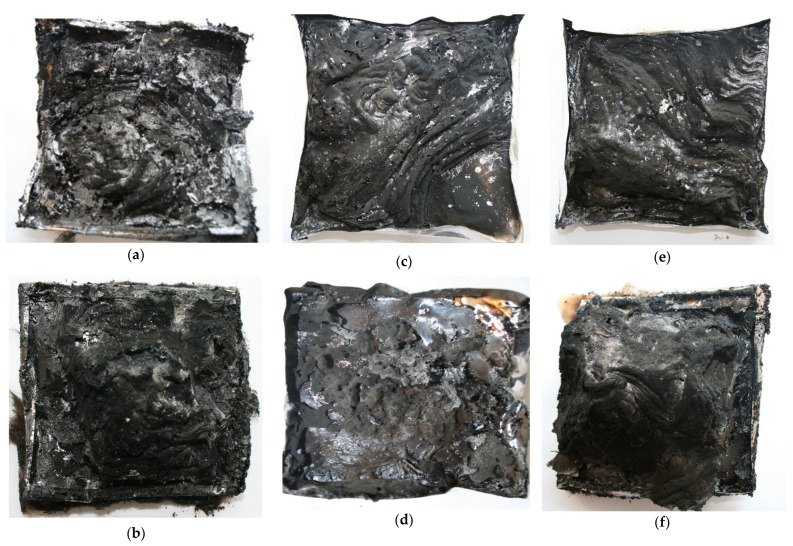
Photographs of the tested materials after the cone calorimetry test: EPUR_1,5 (**a**), EPUUR_TO (**b**), EPUR_2,5 (**c**), EPUR_SO (**d**), EPUUR (**e**), and EPUUR_RO (**f**).

The reduction in the total heat released (THR) from 92 MJ/m^2^ for EPUR_1,5 to 53 MJ/m^2^ for EPUUR_TO, as well as from 86 MJ/m^2^ for EPUUR to 41 MJ/m^2^ for EPUUR_RO, demonstrated incomplete combustion [37]. One of the reasons for the decrease may have been char formation, as confirmed by the photos of EPUUR_TO and EPUUR_RO samples after the CC tests (Figure 3), which caused the fuel release to be reduced. Another reason was the reduction of the combustion efficiency, which was reflected in the values of the effective heat of combustion (EHC). The decrease in EHC due to the addition of TO and RO biopolyols was approximately 45%. In turn, the addition of the biobased polyol SO caused an almost twofold increase in both parameters.

A phenomenon that occurs during a fire is smoke emission, which is a gaseous phase of liquid or solid products of incomplete combustion that are as dangerous as the flame. The total smoke released (TSR) was reduced from 954 m^2^/m^2^ to 437 m^2^/m^2^ as a result of the TO use and from 1134 m^2^/m^2^ to 150 m^2^/m^2^ after applying SO. Only the use of SO contributed to the more than twofold increase in smoke emission. The TSR values decreased once a carbonaceous char was formed, which prevented further degradation of the material and decreased the total amount of smoke [38].

The differences between the tested materials made with the use of biopolyols probably resulted from the structures of these polyols. Vegetable oils, before being used for the production of polyurethanes, must undergo a chemical treatment in order to obtain oligomers (polyols) that are terminated with hydroxyl groups [7]. Vegetable oils must be functionalized, which takes place through double bond reactions, such as epoxidation, hydroformylation, metathesis, or ester bond reactions. Various types of chemicals are used to produce them, such as acids, alcohols, or amine compounds. Additionally, the presented biopolyols have been used in elastomers in other ways.

### 3.3. Analysis of Char Residues after the Cone Calorimeter Test

Important information concerning the flame retardant mechanism of the study materials could be obtained from the char investigations. In Figure 3a–f, photographs of the test material residues after the cone calorimetry test are shown. As presented, the EPUR_1,5 sample had only a small residue yield after the burning process and the char layer was not continuous. The application of RO polyol to the production of EPUUR led to the formation of a protective char layer, which prevented heat transfer and the evolution of decomposition products from the elastomer. It was observed that the carbonaceous char barrier was created on the surface of the sample. The residue had the form of swollen char and was shaped in layers [39]. The increased char yield worked as a physical char barrier, which could block the release of degradation volatiles to feed the flame, and thus, the HRR was slowed down [40,41]. A similar charring effect was also observed only for EPUUR_TO (Figure 3b).

**Figure 4:**
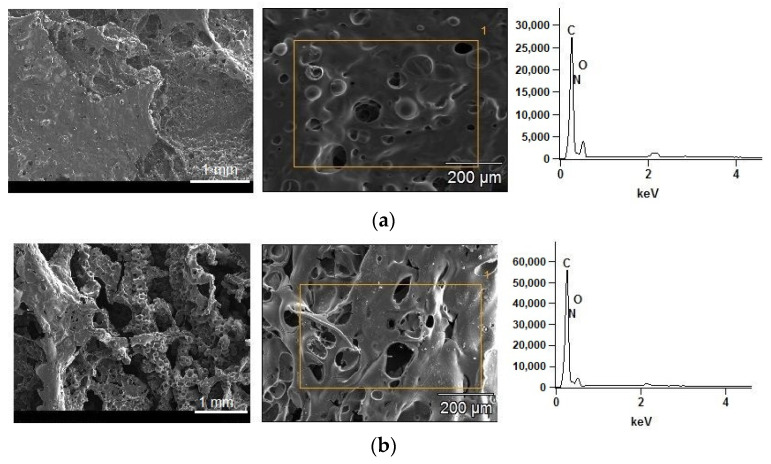

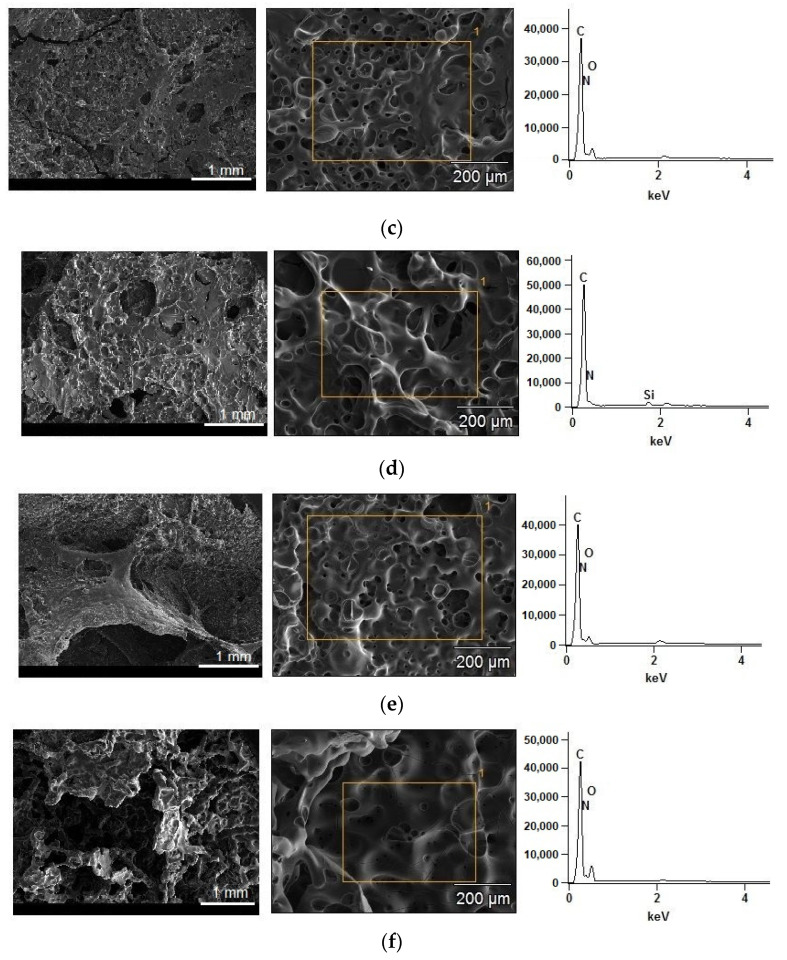
The scanning electron microscopy (SEM) images and energy-dispersive spectroscopy (EDS) analysis of the char residues after the cone calorimeter tests of EPUR_1,5 (**a**), EPUUR_TO (**b**), EPUR_2,5 (**c**), EPUR_SO (**d**), EPUUR (**e**), and EPUUR_RO (**f**).

Figure 4a–f shows the SEM and EDS images with magnifications of 30× and 150×, respectively, to observe the morphologies of the char residue and analyze the elemental composition of the EPURs and EPUURs. It can be seen that the residues of elastomers containing only petrochemical polyol displayed a thin, flat in places, and broken structure because of insufficient char formation. In turn, Figure 4b,f show a three-dimensional structure, which confirmed the occurrence of intumescent char in the case of EPUUT_TO and EPURR_RO. As can be seen in Figure 4d, in the char residues, C, O, N, and Si elements were found. The addition of biobased polyols to the production of EPURs and EPUURs resulted in a higher content of C in the char residues, and the highest growth of C was found in the char of EPUUR_TO, which may have been connected with the TO polyol’s elemental composition (79% carbon, 11% oxygen, 10% hydrogen) [42]. However, due to the porous structure of the char and the different amount of material on which the beam falls, the quantitative comparison of the obtained results may not reflect the actual data. Moreover, Si was observed in EPUR_SO; this was the result of using inorganic and metal catalysts to produce the SO polyol [43].

**Figure 5:**
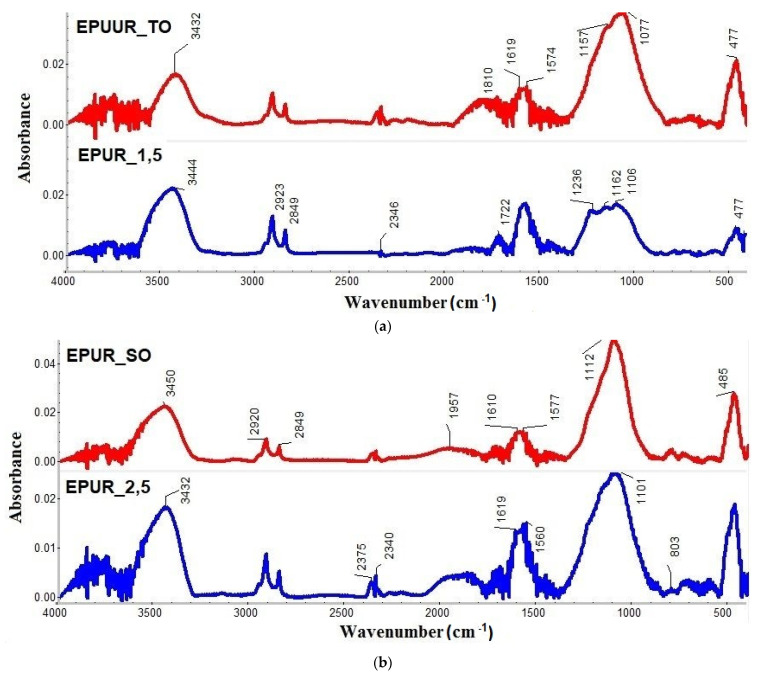

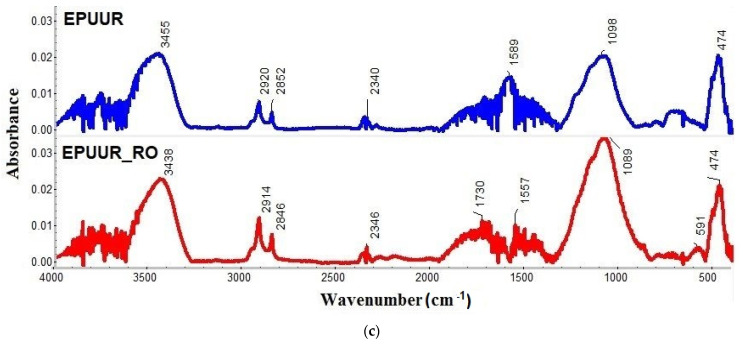
Fourier transform infrared spectroscopy (FT-IR) spectra of the char residues after the cone calorimetry tests of EPUR_1,5 and EPUUR_TO (**a**), EPUR_2,5 and EPUR_SO (**b**), and EPUUR and EPUUR_RO (**c**).

The residual char of the tested polyurethane and poly(urea)urethane elastomers were analyzed using FT-IR after the cone calorimetry tests and their spectra are shown in Figure 5a–c. The peak at 3432–3455 cm^−1^ was related to the N–H stretching vibrations. The peaks at 2914–2923 cm^−1^ and 2846–2852 cm^−1^ corresponded to the vibrations of C–H bonds due to the presence of CH_2_ groups. The absorption peak in the range of 1557–1589 cm^−1^ corresponds to C–C aromatic bonds [44]. The absorption peaks of the C–O bond, corresponding to the formation of hydrocarbons [45], were observed in the range of 1101–1162 cm^−1^ in the FT-IR spectra of the char residues. The small peaks at about 803 cm^−1^ corresponded to C–O bonds in aromatic compounds. For the samples of EPUR_1,5 and EPUUR_RO, the peaks at 1722 cm^−1^ and 1730 cm^−1^ indicated the presence of C=O bond stretching vibrations of carbonyl groups. For the rest of the materials, these bonds had very small absorbances, which is why it was difficult to determine them. For EPUUR_TO, the peak connected with a C–O bond was a toward lower wavenumber (1077 cm^−1^). This was probably related to the presence of urea bonds in this material. The changes in absorbance and displacement of the signals observed in the FT-IR spectra of char residues (Figure 5a–c) are the result of EPURs and EPUURs system modification with biobased polyols. For all materials, an increase in the absorbance of the peak corresponding to the C–O bond in these materials was observed.

### 3.4. Characterization of the Decomposition Products

**Figure 6:**
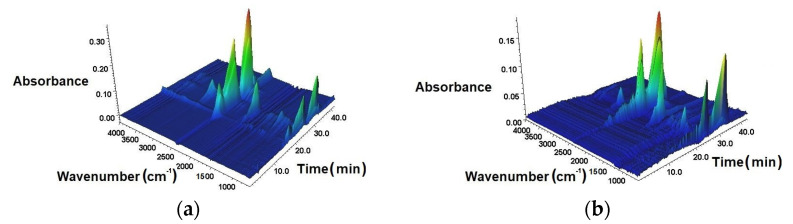

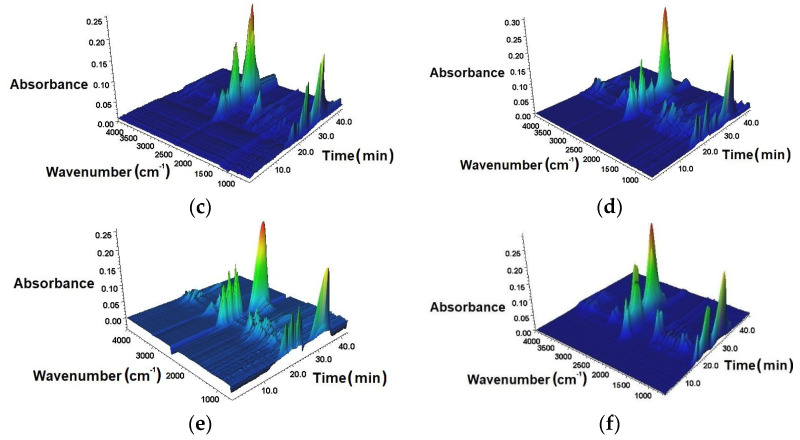
The 3D thermogravimetric analysis (TGA)/FT-IR spectra of EPUR_1,5 (**a**), EPUUR_TO (**b**), EPUR_2,5 (**c**), EPUR_SO (**d**), EPUUR (**e**), and EPUUR_RO (**f**).

**Figure 7:**
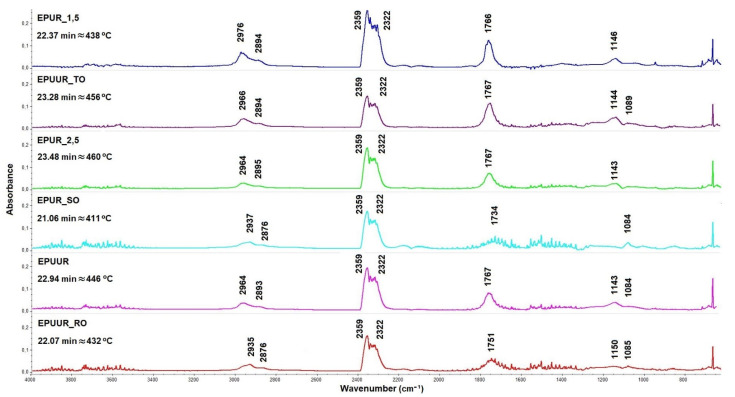
FT-IR spectra of the tested materials at the maximum thermal degradation rate.

In order to study how the biobased polyols exerted the flame retardant effect of EPURs and EPUURs in the gaseous phase, a combination of the TG instrument and FT-IR spectrometer was employed to provide additional information about how the volatile products evolved during their thermal degradation, which could provide insights into understanding the flame retardant mechanisms. Figure 6a–f shows the 3D FT-IR spectra for the gases produced during the thermal degradation of the investigated samples. The FT-IR spectra of the tested materials at the maximum thermal degradation rate are shown in Figure 7. The most volatile products were released in the temperature range of 411–460 °C, which was associated with the samples’ largest loss of weight. The highest intensity for each of the EPUR_1,5, EPUR_2,5, and EPUUR occurred after 22.37 min, 23.48 min, and 22.94 min of measurement, which corresponded to maximum temperatures of 438 °C, 460 °C, and 446 °C, respectively. The introduction of biobased polyols to EPURs and EPUURs resulted in a decrease in the time down to 21.06 min for EPUR_SO, corresponding to 411 °C. The addition of TO polyol to the elastomer matrix led to an increase in the highest intensity from 22.37 min to 23.28 min, which corresponded to temperatures of 438 °C and 456 °C, respectively. For EPUR_SO and EPUUR_RO, decreases in the highest intensities of about 49 °C and 14 °C, respectively, were observed.

The small peaks observed in the wavenumber range of 3500–4000 cm^−1^ were associated with the O–H stretching vibrations from water or hydroxyl compounds [46]. Peaks observed in the range of 2935–2976 cm^−1^ corresponded to the C–H stretching vibrations and the peaks at 2876–2895 cm^−1^ corresponded to the CH_2_ stretching vibrations. These peaks moved toward lower wavenumbers after the addition of biobased polyols to the elastomer matrix. The peaks at 2359 cm^−1^ and 2322 cm^−1^ corresponded to CO_2_ and the absorbance of them decreased after the addition of biobased polyols. Another group of peaks was located at 1734–1767 cm^−1^ and 1143–1150 cm^−1^, which corresponded to C=O and C–O–C, respectively [47]. In the wavenumber range of 1084–1089 cm^−1^, a peak attributed to the stretching vibrations of C–O was observed. These peaks had low absorbance.

After the addition of biobased polyols to elastomers, the absorption intensity of C–H, –CH_2_, C=O, C–O–C, and C–O were weakened, and the rest of the C, as well as the aromatic compounds, remained in the condensed phase and took part in the formation of the char residue [42]. Above the temperature of 550 °C, the degradation of the pre-char took place and the peaks that corresponded to CO_2_ had higher intensities, and new peaks corresponding to C–O bonds appeared. The introduction of the TO polyol resulted in a decrease in the absorption intensities of the mentioned peaks, which confirmed the results obtained in the cone calorimeter test (Table 4).

**Figure 8:**
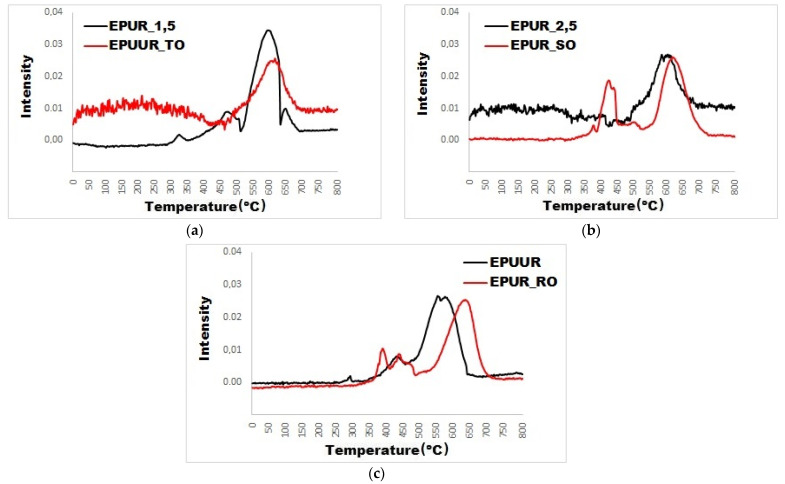
Absorbance intensity versus the temperature of the carbon monoxide decomposition products during thermal decomposition (**a**–**c**).

**Figure 9:**
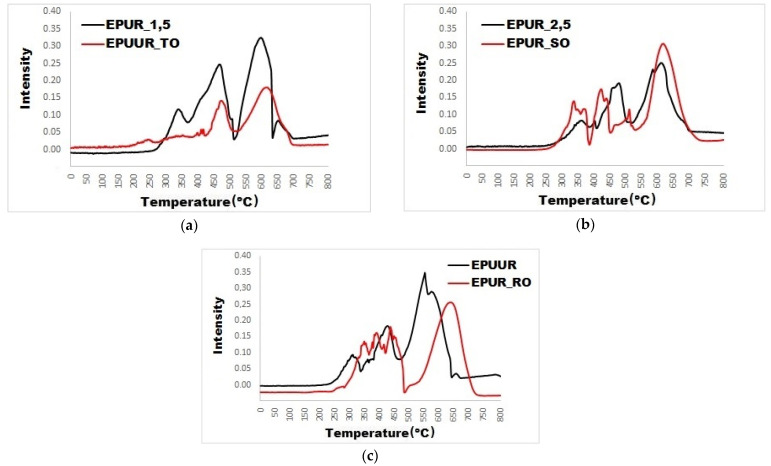
Absorbance intensity versus the temperature of the carbon dioxide decomposition products during thermal decomposition (**a**–**c**).

For a more accurate analysis of the impact of the addition of biobased polyols to elastomers, the absorbance intensities of the distinct decomposition products are shown in Figure 8a–c and Figure 9a–c. Carbon monoxide is a toxic gas and is the chief factor of death in fire disasters. The addition of biobased polyols did not affect the release of carbon monoxide (Figure 8a–c), and the addition of TO even reduced it. Since some of the carbon stays in the condensed phase, the reduction in the gaseous products is related to the char formation, as well as the fact that char acts as a physical barrier for the released products of incomplete combustion. The TO polyol can play an important role in the application of fire safety materials.

TO is a by-product of cellulose production. It is known from [48] that cellulose and lignin promote char formation. TO-based polyol is a mixture of different fatty (C12–C22) and rosin acids (C20), such as fatty acid–oleic acid (C_18_H_34_O_2_) and rosin acid–abietic acid (C_20_H_30_O_2_). The use of diethanolamine in the production of the TO-based polyol is also an important aspect, which may reduce the reaction to fire. The functionality of the used TO polyol was 2.15, which led to branching in the EPUUR. The use of the TO-based polyol as a chain extender in the production of EPUUR probably promoted the formation of physical and chemical bonds which, during combustion, favored the formation of the char. In turn, the formation of the char during smoking, which acted as a barrier, hindered the heat transfer, and thereby, reduced the release of flammable volatile products.

Furthermore, it was observed that the release of the carbon dioxide was decreased after the addition of TO polyol (EPUUR_TO) to the elastomer (EPUR_1,5) (Figure 9a) and it had a lower intensity than with EPUR_SO and EPUUR_RO, which was observed in the FT-IR spectra.

## 4. Conclusions

In this study, the influence of biobased polyols, such as TO-based polyol, SO-based polyol, and RO-based polyol, on the burning behavior and fume emissions of EPURs and EPUURs were investigated. The thermal stability, burning behavior, and smoke emissions of the produced materials were evaluated using TGA in nitrogen or air atmospheres and cone calorimetry tests. The released gases were identified using TGA/FT-IR.

The results from the thermogravimetric analysis and derivative thermogravimetric analysis in a nitrogen atmosphere demonstrated that the addition of TO polyol to EPURs delayed the maximum degradation process of the material, and in air, improved the char yield. Adding the TO and RO polyols led to a sharp drop in HRR values. The addition of TO and RO polyols led to an evolving nonflammable gas phase and the formation of a protective char, which prevented heat and mass transfer. This was probably the effect of the chemical treatment of these oils in the production of the biobased polyols. In the case of materials with TO polyol, the emission of carbon monoxide was the lowest and the total carbon dioxide was almost 40% lower than from the reference material, which was due to its chemical structure.

The presented results are an introduction to further research on the impact of these polyols on the flammability of new bioelastomers. It has been observed that changing the polyol in terms of which flexible segments are formed significantly influences the properties of the obtained material, changing its thermal properties and ability to burn. The functionality and molecular weight of the biopolyol used are probably important, as it affects the degree of phase separation, and thus, the properties of the elastomers obtained.

In general, the addition of biobased polyols to polyurethane and poly(urea)urethane elastomers appear to be a promising solution for industrialization, where high thermal stability and a reduction in flammability are required and it is important to follow the trends of sustainable development.

## Figures and Tables

**Table 1:** Characteristics of the prepared materials.

Sample Designation	Polyol—Amount (mol)	Isocyanate—Amount (mol)	Chain Extender—Amount (mol)	HS (wt%)
EPUR_1,5	PEA—1	MDI—1.55	EG—0.5	17.6
EPUUR_TO	PEA—1	MDI—1.55	TO—0.5	21.1
EPUR_2,5	PEA—1	MDI—2.55	EG—1.5	23.7
EPUR_SO	SO—1	MDI—2.55	EG—1.5	33.2
EPUUR	PEA—1	MDI—1.55	DYDI—0.5	24.5
EPUUR_RO	PEA—0.5/RO—0.5	MDI—1.55	DYDI—0.5	29.6

DYDI—dicyandiamide, EG—ethylene glycol, HS—hard segment, MDI—4,4′-diphenylmethane diisocyanate, PEA—poly(ethylene adipate), RO—rapeseed oil, SO—soybean oil, TO—tall oil.

**Table 2:** Thermal properties found using the TG and DTG analyses of the tested materials (in a nitrogen atmosphere).

Sample Designation	T_5%_	T_1_	m_1_	T_2_	m_2_	T_3_	m_3_	T_4_	m_4_	T_5_	m_5_	R_800_
	(°C)	(°C)	(%)	(°C)	(%)	(°C)	(%)	(°C)	(%)	(°C)	(%)	(%)
EPUR_1,5	293	-	-	323	18.2	376	45.4	414	26.6	-	-	7.3
EPUUR_TO	264	-	-	-	-	387	36.9	-	-	430	50.4	4.4
EPUR_2,5	303	-	-	328	13.0	-	-	-	-	440	77.3	7.5
EPUR_SO	294	-	-	319	16.6	-	-	415	53.5	476	21.3	5.4
EPUUR	289	298	9.5	-	-	-	-	400	76.8	-	-	9.1
EPUUR_RO	284	304	10.9	-	-	375	73.3	-	-	-	-	7.9

T_5%_—temperature at 5% mass loss; T_i_ and m_i_—degradation stage i’s temperature and weight loss, respectively; R_800_—residue at 800 °C.

**Table 3:** Thermal properties found using the TG and DTG analysis of the tested materials (in an air atmosphere).

Sample Designation	T_5%_	T_1_	m_1_	T_2_	m_2_	T_3_	m_3_	T_4_	m_4_	T_5_	m_5_	T_6_	m_6_	T_7_	m_7_	R_800_
	(°C)	(°C)	(%)	(°C)	(%)	(°C)	(%)	(°C)	(%)	(°C)	(%)	(°C)	(%)	(°C)	(%)	(%)
EPUR_1,5	297	-	-	322	16.3	-	-	400	28.4	442	31.2	-	-	561	22.7	0.9
EPUUR_TO	292	216	11.0	-	-	-	-	399	15.1	432	51.7	-	-	550	19.4	2.2
EPUR_2,5	304	-	-	324	15.0	-	-	401	28.0	444	33.0	-	-	559	22.2	0.7
EPUR_SO	279	199	1.7	316	17.9	361	8.2	384	14.5	416	18.9	468	14.4	570	23.4	0.6
EPUUR	310	-	-	325	8.7	-	-	395	27.9	439	38.0	-	-	583	22.2	1.1
EPUUR_RO	297	-	-	326	12.9	372	23.7	419	17.3	-	-	469	19.2	579	24.2	0.2

**Table 4:** Summary of the cone calorimeter data.

Sample Designation	TTI	Av-HRR	MAHRE	PHRR/tPHRR	THR	EHC	TSR
	(s)	(kW/m^2^)	(kW/m^2^)	(kW/m^2^s^1^)	(MJ/m^2^)	(MJ/kg)	(m^2^/m^2^)
EPUR_1,5	41 ± 6	262 ± 7	407 ± 21	7.2 ± 1	92 ± 8	22 ± 1	954 ± 19
EPUUR_TO	81 ± 10	95 ± 17	170 ± 28	2.3 ± 0	53 ± 9	9 ± 1	437 ± 44
EPUR_2,5	38 ± 1	261 ± 60	354 ± 21	7.5 ± 1	68 ± 4	18 ± 1	740 ± 25
EPUR_SO	38 ± 2	471 ± 26	613 ± 28	23. 5 ± 7	116 ± 3	30 ± 1	2367 ± 35
EPUUR	36 ± 4	280 ± 26	398 ± 39	7.8 ± 0	86 ± 3	22 ± 0	1134 ± 92
EPUUR_RO	33 ± 0	73 ± 5	180 ± 10	4.0 ± 0	41 ± 2	10 ± 0	150 ± 10

TTI—time to ignition, Av-HRR—average heat release rate, MAHRE— maximum average rate of heat emission, PHRR—peak heat release rate, tPHRR—time to peak heat release rate, THR—total heat released, EHC—effective heat of combustion, TSR—total smoke released.

## Data Availability

The data presented in this study are available on request from the corresponding author.
